# Do political connections facilitate or inhibit firms’ digital transformation? Evidence from China’s A-share private listed companies

**DOI:** 10.1371/journal.pone.0302586

**Published:** 2024-05-07

**Authors:** Xingye Jin, Tao Li, Yupeng Shi, Mingrui Zhang

**Affiliations:** 1 School of Economics, Central University of Finance and Economics, Beijing, China; 2 China Center for Internet Economy Research, Central University of Finance and Economics, Beijing, China; Nanjing Audit University, CHINA

## Abstract

Given the advent of the digital era, digital transformation has become necessary for enterprise development. Political connections are the most important resources for enterprise development in most countries. However, the impact of political connections on corporate digital transformation has yet to be verified. This study uses ERNIE, a large language model, to construct a measurement of corporate digital transformation from the perspective of digital technology application through a textual analysis of the annual reports of A-share privately listed companies from 2008 to 2020 and analyzes the impact of political connections on corporate digital transformation and its mechanism of action. The findings demonstrate that political connections have a significant inhibitory effect on corporate digital transformation. This conclusion still holds after a series of robustness and endogeneity tests. The mechanism analyses demonstrate that political connections primarily affect corporate digital transformation through three mechanisms: weakening risk, inhibiting innovation, and enhancing resource crowding. We theoretically expand the understanding of the economic impact of political connections and provide new ideas for accelerating enterprise digital transformation from the perspective of policy makers.

## 1. Introduction

In recent years, Digital economy is becoming more and more important. According to calculations from the China Academy of Information and Communications Technology, in 2022, the digital economy of 51 major economies worldwide reached $41.4 trillion, increasing $2.9 trillion compared to that of previous year. The digital economy has become an important engine driving global economic growth during the pandemic and a transformative force in reshaping the global value chain in the postpandemic era. In recent years, major countries and economies such as the United States, the European Union, and Japan have successively issued relevant strategic policies, according to their own industrial characteristics, factor endowments, development goals, etc., to strengthen the deployment of advanced technologies such as microelectronics, automation, and information in the manufacturing industry and actively promote enterprise digital transformation (DT) and development. From the enterprise perspective, to adapt to the transformation of traditional production models and to achieve sustainable development, enterprises are also actively carrying out DT. Enterprise DT is the process of introducing digital technologies, such as artificial intelligence, blockchain, big data, and cloud computing, into production management, organizational operations, research, development innovation, and sales services. This transformation process reduces repetitive labor or replaces traditional technology with advanced technology through the application of digital technology [1]. The 2022 report of the United Nations Industrial Development Organization showed that manufacturing enterprises that use high-level digital technology also perform much better than other enterprises in terms of various economic indicators, such as monthly sales, annual profits, and employment rates [2].

However, DT of enterprises is still in its early stages, and the transformation process is not smooth. According to a survey carried out by Harvard Business Review, DT risk is the first concern of corporate directors. However, 70% of all DT initiatives do not reach their goals. In addition, $900 billion out of the $1.3 trillion spent on DT last year was estimated to be wasted. Enterprises usually need to utilize many resources to carry out DT. To successfully achieve transformation goals, most enterprises seek external assistance. Among numerous external resources, the government, as an indispensable entity in DT, is also one of the most important external resources owned by enterprises and profoundly affects their decision-making behavior [3, 4]. Many national governments and multilateral organizations have indeed introduced strategic foresight studies to ground their long-term policies [1]. Political connections are widespread in both developed and developing countries [5]. Although the political resources of different enterprises and the closeness of their relationships with the government are different, generally, constructing a good relationship with the government is still commonly pursued by enterprises. Many enterprises facing resource constraints seek to build political connections in the DT process [6]. On one hand, political connections can help enterprises reduce political uncertainty, enabling them to obtain substantial support in information, technology, talent, funding, and other aspects during the DT process and achieving strategy and resource sharing among enterprises [7, 8]. On the other hand, the decision-making behavior of enterprises with close political connections is also more susceptible to government influence, participating more in nonproductive activities, weakening corporate performance, and possibly not fully achieving optimal resource allocation [9, 10]. Therefore, the impact of political connections on enterprise DT is a topic that needs to be verified. Existing research on the factors influencing enterprise DT has focused mostly on external factors such as peer effects and the business environment [11], as well as internal factors such as entrepreneurial spirit and talent reserves [12, 13]. However, existing research has not focused on the possible impact of the important factors of the government—business relationship. In the context of the rapid advancement of DT, considering the importance of political connections and the universality of enterprises actively building political relationships, studying the impact of corporate political business relationships on DT and clarifying their internal impact mechanisms have important theoretical and practical significance.

Based on the above considerations, we take Chinese A-share listed companies as an example to study the relationship between political connections and enterprise DT. China is the research country because of the relatively rapid development of its digital economy. According to the China Digital Economy Development Report (2022), the country’s digital economy was expected to be 45.5 trillion yuan in 2021, accounting for 39.8% of GDP and reflecting a year-on-year growth rate of 16.2%. In addition, as a transition country, government involves a lot in corporate behavior in China, which provides a good environment for us to study the relationship between political affiliation and corporate DT. Specifically, we focus on the following two questions. First, is there an impact of political connections on corporate DTs? We choose private enterprises because the political connections of most SOEs are “natural” rather than the consequences of corporate behavior [14]. In contrast, private firms’ pursuit of building political connections is more purposeful, and they pay more attention and invest more resources in building and maintaining such “relationships” [15]. Second, if political connections significantly impact corporate DT, what are the underlying mechanisms?

We use the Enhanced Representation through Knowledge Integration (ERNIE) pretraining model to construct DT measurement indices, the political ties of the enterprise chairperson or general manager and enterprise on-the-job consumption expenditures as a percentage of operational revenue to measure political connections. We analyze the influence of political connections on corporate DT and its mechanism of action, providing systematic answers to the above two questions. The estimation results of panel regressions based on two-way fixed effects and various robustness and endogeneity tests indicate that political connections have an inhibitory effect on corporate DT. Additionally, a heterogeneity analysis demonstrates that this inhibitory effect is more pronounced among strongly competitive firms, firms with lower revenue growth rates, non-high-tech firms, and firms whose municipal party secretaries at the prefecture level changed during the year. The mechanism analysis demonstrates that the inhibitory effect of political connections on corporate DT works through three main mechanisms: risk-weakening, innovation-inhibiting, and resource-crowding-out effects.

Compared with the previous literature, our study contributes to the literature in two ways. First, we complement the research on the impact of political connections on corporate DT. Currently, an increasing number of papers are discussing on the impact of political connections on firm performance and behavior, such as political connections on financing [14, 16, 17], tax breaks [5, 18], investments [19], mergers and acquisitions (M&A) [20], and other behaviors. Only a few studies have linked political connections and firms’ DT. Through this article, we add to the existing related research from the perspective of DT by exploring the impact and mechanism of political connections on corporate DT, offering a new perspective for examining whether political connections are an “enabling hand” or “intervening hand”. We use data from Chinese listed enterprises and measure the level of DT based on annual reports. This approach is more comprehensive and objective. Furthermore, by using instrumental variable methods, high-order fixed effects and propensity score matching (PSM), possible endogeneity problems can be mitigated to a certain extent, increasing the robustness and reliability of the research findings.

Second, the mechanism through which political connections influence enterprises’ DT is explored in depth. We propose risk-weakening, innovation-inhibiting, and resource-crowding-out effects of political connections on private enterprises’ DT and explore the mechanism of the inhibitory effect of political connections on private enterprises’ DT through theoretical analysis and mechanism testing.

## 2. Literature review and theoretical analysis

### 2.1 Research related to political connections

Enterprises’ political connections are informal and special ties between enterprises and government departments or individuals with political power, particularly the ability to obtain resources from government departments; this ability represents enterprises’ most important social capital and institutional environment [20]. In China, the government is a major participant in economic activities, and understanding government behavior is key to understanding China’s economic development and the behavior of economic agents. Because governments at all levels in China play an important role in attracting investments, engaging in industrial policy-making, managing land, and developing tax subsidies, firms can build close government—business relationships to improve trust in government entities and obtain more resources [21].

Political connections significantly affect numerous firm behaviors, such as financing, investments, and M&As, and can reduce financing and issuance costs [5, 10, 14, 16–19]. For example, the party membership of Chinese private entrepreneurs can help firms obtain loans from banks or other financial institutions more easily and build more confidence in the judicial system, especially in regions with less marketization and less property rights protection [16]. Political—business ties can reduce the cost of obtaining bank loans, and banks typically do not impose restrictions on these firms in their borrowing contracts regarding liquidity requirements or capital expenditures [22]. In addition, politically connected firms have lower costs when issuing stock [17].

Political connections also promote corporate diversification and increase investments. Government agencies such as the U.S. Federal Trade Commission determine whether corporate M&A activities can be carried out successfully; therefore, the likelihood of firms undertaking M&A activities increases when existing government personnel or regulators are hired as corporate board members [20]. Houston et al. (2014) [23] found that firms with strong political connections can learn about changes in tax policies and enforcements in advance, facilitating tax avoidance. Kim and Zhang (2016) [24] used firms that received bankruptcy protection and restructuring from 1998 to 2010 as examples and found that those with strong political connections were more likely to resolve their difficulties through out-of-court restructurings because such political connections signaled to the market that the firm value was likely to recover quickly. However, few studies have been conducted on how political connections affect corporate DT. We believe that including DT in the scope of the research on the influence of political connections on the behavior of listed enterprises help us understand the path of corporate growth in the context of the rapid development of the digital economy and deepen the understanding of the economic impact of political connections.

### 2.2 Possible impact of political connections on corporate digital transformation

As private firms face the trade-off between enhancing their internal capabilities and seeking external government assistance for their development, the impact of political connections on firms has both facilitating and inhibiting effects, which can promote firm development by enhancing firm value, increasing performance, improving firm financing ability, and breaking down industry barriers [25–27]. This impact can also inhibit firm development by worsening long-term firm performance, impeding innovation, and increasing social burdens [28–30]. Therefore, from a theoretical perspective, the impact of political connections on private firms’ digital transformation is uncertain.

#### 2.2.1 The logic that political connections may facilitate corporate digital transformation

First, close political connections provide private enterprises with greater financial support. Digital transformation requires private enterprises to invest considerable resources, and political connections can alleviate the lack of capital that enterprises may face when pursuing digital transformation. Many studies have demonstrated that political connections can alleviate private enterprises’ financing constraints by providing more favorable loan terms, extending loan maturities, and promoting targeted issuances [16, 31, 32]. In the financial market, information asymmetry exists between the supply and demand of funds, and capital providers such as banks have difficulty distinguishing between superior and inferior enterprises. Generally, banks and other capital providers believe that enterprises with political connections are more creditworthy; therefore, they do not need to worry about the risk of debt default. Thus, they tend to provide credit channels with longer maturities and lower or more favorable interest rates. In addition, political connections bring many special subsidies or tax breaks to affiliated enterprises [16, 18], directly providing the funds needed for DT.

Second, close political connections enable private firms to gain an information advantage. Under the premise that DT requires substantial investments, enterprises must understand the government’s focus areas and DT policies. Political connections have a government bias effect on private enterprises [33], which helps them obtain first-hand policies or information. By establishing political ties, firms can know in advance the government’s key development direction and schedule regarding DTs, which guides them to invest precisely in key development areas. In addition, the existence of political connections reduces the probability that corporate executives are dismissed for poor performance [34], making companies more comfortable and patient with engaging in DT activities that require long-term sustained investments.

Based on the above analysis, we propose the following hypotheses:

H1: Political connections facilitate digital transformation of private enterprises.
H1a: Political connections enable private enterprises to obtain more financial support, which in turn promotes their digital transformation.
H1b: Political connections enable private enterprises to gain information advantages, which in turn promotes their digital transformation.

#### 2.2.2 The logic that political connections may inhibit corporate digital transformation

First, having close political connections weakens private firms’ willingness and ability to take risks, creating a lack of incentive to undertake DTs. On the one hand, private firms are generally risk averse at the investment level [35], and when firms establish close political connections, they are more likely to devote political resources to low-risk, high-return business areas, which in turn reduces the variety of financial and managerial resources invested in creative production activities [36, 37]. As the DT cycle is long, domestic enterprises generally face the situation that they dare not transform because of the long investment time needed for DT; therefore, private enterprises are likely to have a reduced preference for risky and uncertain projects such as DTs. On the other hand, political connections make it easier for private enterprises to obtain government subsidies [33], lower interest rates, or longer-term bank loans [16, 21], creating the tendency for enterprises to adhere to the rules and “rest on their laurels”, reduce their willingness to take risks and have less urgency for DT. However, the urgency of DT is insufficient. In addition, compared with SOEs, private firms have established more fragile political ties and are more susceptible to institutional factors, such as official turnover and anticorruption, resulting in more cautious corporate decisions.

Second, close political connections may have a disincentive effect on private firms’ technological innovation, making them less motivated to engage in innovation. Whether firms can successfully achieve DT relies more on their innovation capabilities. With the rapid development of the digital economy, firms have a stronger incentive to focus their innovative R&D activities on DT projects. Private enterprises with close political connections generally face relatively weak competitive market environments [13]. In general, listed private enterprises are an important source of tax revenue and employment for local governments. When facing competition between local and foreign enterprises, many local governments adopt local protectionism, such as directly assisting in the development of local enterprises with close political and business ties by providing substantial subsidies and large purchase orders, resulting in weaker market competition and weaker incentives to innovate. On the other hand, as private firms become richer in political resources, they may seek to improve their performance through nonproductive activities, such as rent-seeking, rather than innovative activities, and their willingness to innovate actively decreases. In addition, political—business ties can reduce internal inventors’ willingness to participate in innovation, causing innovation to decline.

Third, close political connections consume many private enterprise resources and have a “crowding-out” effect on investments in enterprise DT. On the one hand, close political connections incur many nonproductive expenses. Compared with SOEs, private enterprises build political connections to change their identity from “outsiders” to “insiders,” and enterprises often need to use many resources in this process, such as excessive executive consumption and other nonproductive expenditures [28, 29, 38]. Doing so leads to attrition in enterprises’ normal production and operations and crowds out resources to be used for DTs. On the other hand, political connections increase the social responsibility burden on private enterprises. Due to the prevalent political tournament promotion system for local government officials in China, in this context, private enterprises with political connections tend to take on additional social responsibilities to cater to government requirements [36], such as absorbing more local labor to provide employment, actively participating in social charity or donations, undertaking economic growth or taxation tasks, and thus “crowding-out” investments in corporate DT.

Based on the above analysis, we propose the following hypotheses:

H2: Political connections inhibit the digital transformation of private enterprises.
H2a: Political connections weaken the risk-taking ability of private enterprises, which in turn hinders their digital transformation.
H2b: Political connections inhibit private enterprise innovation, which in turn hinders the digital transformation of enterprises.
H2c: Political connections increase the resource depletion of private enterprises, thereby inhibiting their digital transformation.

## 3. Study design

### 3.1 Data sources and sample selection

We use data on A-share privately listed enterprises that trade on the Shanghai Stock Exchange (SSE) and Shenzhen Stock Exchange (SZSE) from 2008 to 2020. We treat the data as follows. First, we exclude enterprises in the financial and insurance industries. Second, we exclude enterprises with special treatment in the ST and *ST categories. Third, we exclude enterprises with observations not more than two years old and with too many missing indicators. Fourth, to minimize the influence of outliers, we treat all continuous variables with 1% tailing. After processing, unbalanced panel data for 2,349 private enterprises with 18,622 observations are obtained. Annual report data are obtained from the official websites of the SSE and SZSE, and financial and governance data are obtained from the Wind and CSMAR databases.

### 3.2 Variable measures

#### 3.2.1 Dependent variables

The explanatory variable in this study is the level of DT of private enterprises. Three main metrics are used in the literature. The first is the questionnaire method. The questionnaire included questions related to corporate DTs, and factor or principal component analyses were used to measure the level of DT according to the questionnaire [39, 40]. However, questionnaire data are susceptible to sampling methods and are highly subjective, and their accuracy needs to be verified. The second is the objective indicator measurement method. Examples include whether enterprises use e-mail or have their own websites [41], whether they use CAD [42], the proportion of specific intangible assets related to the digital economy [43], and the proportion of employees who frequently use computers. However, given the continuous development of the digital economy, describing corporate DT with objective indicators is not easy; therefore, these measures are not sufficiently precise. The third method is the word frequency method, which determines DT keywords based on important documents and uses the frequency at which keywords appear in enterprises’ annual reports to measure the degree of DT [44]. However, this method can result in discriminatory errors. Given the continuous expansion of the boundaries of the digital economy, exhausting keywords related to DT are difficult. Moreover, the presence of keywords in sentences does not mean that enterprises have engaged in DT. For example, many keywords appear in sentences describing the macro development context; therefore, measuring the importance of a word simply by its frequency is not sufficiently comprehensive.

To solve the possible problems of the word frequency method in identification and measurement, we follow the method of Jin et al. (2024) [45]. They use the supervised machine learning ERNIE model to measure corporate DT from the perspective of using digital technology. The main content of the supervised machine learning model involves manually constructing the training set, testing the validity of the training set through the validation set, continuously iterating to improve training accuracy, calculating the training results through the test set, and using them for the final index calculation.

**Fig 1:**
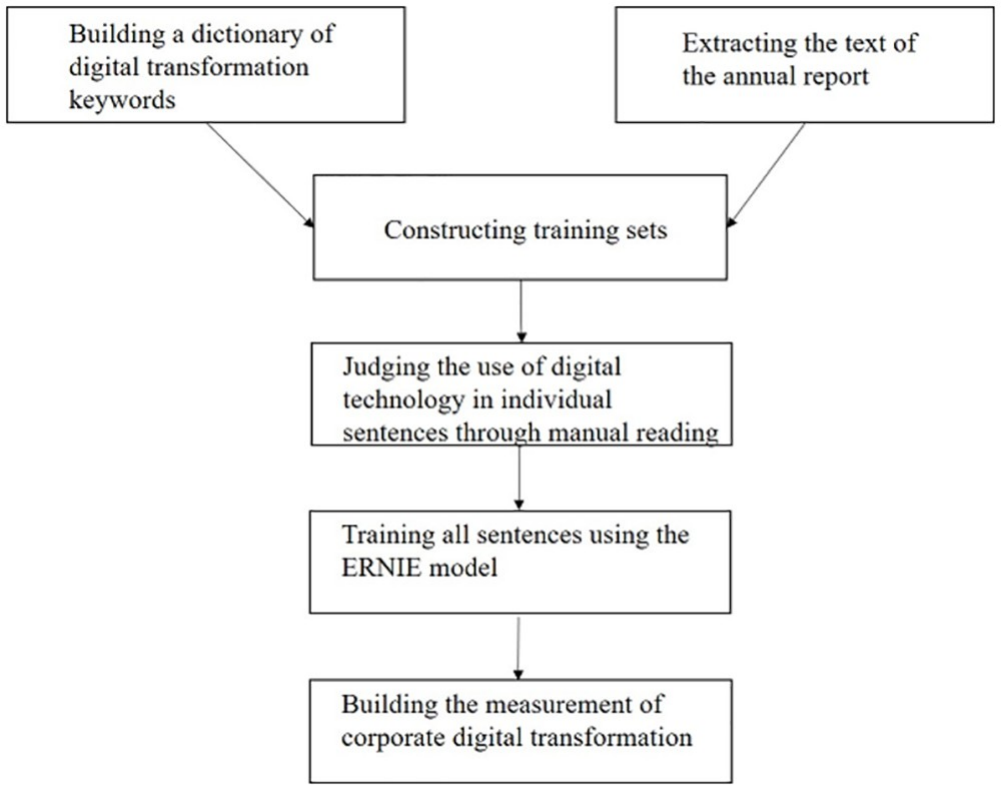
Construction process of corporate digital transformation.

Fig 1 shows the construction process. First, a dictionary of keywords related to digital technology utilization is constructed. Because the uneven distribution of digital-technology-related sentences in corporate annual reports decreases the efficiency of manual reading and labeling, Jin et al. (2024) narrows the scope of random reading by selecting keywords to build a dictionary and finding texts more likely to describe digital technology [45]. By reading annual reports of listed companies, digital economy-related policy documents, and domestic and foreign research literature on the digital economy, Jin et al. (2024) identify six digital technology categories—big data, cloud computing, Internet of Things, blockchain, mobile internet, and artificial intelligence—and use them as seed words [45]. Jin et al. (2024) also use the word2vec word vector algorithm in machine learning from management discussion and analysis (MD&A) texts from annual reports to find words with similar meanings to the seed words and construct a dictionary of keywords used in corporate digital technology [45].

Second, a training set is constructed. The authors manually tag all sentences in the text, mark whether the sentences are related to digital technology and the kind of digital technology to which they are related, and construct a training set to construct a machine learning model.

Third, the ERNIE model is used to train the training set, and then all the sentences in the text are classified to determine on a sentence-by-sentence basis whether each one implies that the enterprise uses digital technology and, if so, the type. According to a manual comparison, the accuracy of the ERNIE model for annual report text classification exceeds 80%.

Fourth, based on the classification results of the model, we construct the following index: the proportion of words in sentences that imply that the enterprise is or will use the six types of digital technologies in the MD&A section of the annual report, which measures the level of enterprise DT. In addition, as a supplement to the baseline measurement, we also use the proportion of words in sentences, which implies that enterprises currently use the six types of digital technology (DT_now), and a dummy variable indicating whether the enterprise uses digital technology (DT_dummy) as an explanatory variable.

#### 3.2.2 Explanatory variables

The explanatory variable in this study is private enterprises’ political connections (Rel). The measurement of political connections is divided into subjective and objective approaches: subjective measures include questionnaires or case interviews, which measure the closeness of political connections by designing questions on the relationship between enterprises and government departments and by using the subjective feelings of enterprise managers; objective measures tend to measure the political ties of enterprise executives. Many studies have measured political connections based on whether board members have experience in government departments or the proportion of executives with a political background [18, 25, 33]. In addition, several studies have measured political connections through several corporate financial or corporate governance indicators. For example, some studies have used the proportion of on-the-job consumption expenditures, such as business hospitality and travel expenses, to main business revenue to measure political connections [46, 47]. This is because private enterprises and government departments lack stable contact mechanisms at the institutional level. Moreover, constructing and maintaining political connections usually requires private enterprises to take the initiative to increase nonproductive expenditures (e.g., hospitality and travel expenses) to maintain government—business relationships.

Combined with the previous analysis, we measure political connections in two ways. First, from the perspective of corporate executives, if such executives have served or are currently serving in party committees and governments at all levels or the military or have been elected as deputies to the National People’s Congress or members of the Chinese People’s Political Consultative Conference at all levels, the firm is considered to have close political relationships (Rel_PC), which take the values of 1 and 0 in the opposite direction [33]. Referring to common practices in the literature [18], we also consider the political status of the chairperson and general manager. Based on the biographies of the chairpersons and general managers disclosed by listed companies, we compile information on whether the companies have political connections, and the missing information is supplemented by Baidu.

In addition, we define the variable for private enterprises’ political connections (Rel_PCLevel) according to the different administrative levels of the organizations at which the executives work (the county level and below, the prefecture and department level, and the provincial-ministerial level and above) to examine the impact of the closeness of political connections at different administrative levels on corporate DT.

Second, from the perspective of the enterprises themselves, as corporate on-the-job consumption is an important means for enterprises to establish close ties with the government, we add up accounts in the breakdown of corporate overhead that are closely related to executives’ on-the-job consumption, such as travel expenses and business hospitality. The amount of on-the-job spending as a percentage of operating revenue (Rel_ZD) is used to measure the closeness of political connections. The greater the proportion of on-the-job spending is, the stronger are the political connections [46].

#### 3.2.3 Other control variables

Table 1 shows the variables we used. We select the following control variables: enterprise size (Size), age (Lnage), cash flow (Cash), liability-to-asset ratio (Lev), return on assets (ROA), growth rate of operating revenue growth (Growth), shareholding ratio of the top ten shareholders (Top10), and level of internet development in the company’s location (Internet).

**Table 1:** Variable descriptions.

Variable Type	Symbols	Variable Name
Dependent variables	*DT*	Digital transformation level
	*DT_now*	
	*DT_dummy*	
Explanatory variables	*Rel_ PC*	Political connections
	*Rel_ PCLevel*	
	*Rel_ZD*	
Control variables	*Size*	Enterprise size
	*Lnage*	Business age
	*Cash*	Company cash flow
	*Lev*	Gearing ratio
	*ROA*	Net profit margin
	*Growth*	Growth rate
	*Top10*	Shareholdings of top 10 shareholders
	*Internet*	Number of internet broadband access ports divided by the average resident population in that year

### 3.3 Descriptive statistical analysis

Table 2 reports the results of the descriptive statistics of the variables. Due to some listed companies not disclosing travel and other entertainment expenses, variable Rel-ZD has some missing values. To address this problem, we assign the missing value to 0. Regarding DT indicators, most private enterprises have undergone DT, with the DT_dummy averaging 0.89; the level of DT varies across enterprises and is on average 0.07 with a standard deviation of 0.089. Regarding political connections, Rel_PC averages 0.34, indicating that close political connections are still a private enterprise’s “scarce resource”. The distribution of other control variables also has high dispersion.

**Table 2:** Descriptive statistics.

Variables	Observations	Average value	Standard deviation	Minimum value	Maximum value
Dependent variable: digital transformation					
*DT*	18622	0.07	0.090	0.00	0.41
*DT_now*	18622	0.04	0.061	0.00	0.29
*DT_dummy*	18622	0.89	0.311	0.00	1.00
Explanatory variable: political connections					
*Rel _PC*	18622	0.34	0.474	0.00	1.00
*Rel_PCLevel*	18622	0.76	1.158	0.00	3.00
*Rel_ZD*	18622	0.01	0.195	0.00	23.08
Control variables					
*Size*	18622	7.93	1.116	5.58	11.49
*Lnage*	18622	2.86	0.316	2.08	3.56
*Cash*	18622	0.05	0.072	-0.17	0.26
*Lev*	18622	0.38	0.203	0.04	0.92
*Growth*	18622	0.14	0.305	-0.52	1.66
*ROA*	18622	0.07	0.074	-0.25	0.29
*Top10*	18622	0.59	0.152	0.22	0.89
*Internet*	18622	0.54	0.258	0.06	0.99

Table 3 reports the distribution of Rel_PCLevel at the political connection level, where high-level political connections (provincial-ministerial level and above) account for the majority (46.6%) of the sample firms with close political connections.

**Table 3:** Distribution of political connections.

	*Rel_PCLevel = 1*	*Rel_PCLevel = 2*	*Rel_PCLevel = 3*	Total volume
*Rel_PC = 1*	1464	1928	2956	6348
Percentage of	23.0%	30.4%	46.6%	100%

To analyze how political connections affect corporate DT, the sample is classified according to whether they have political connections (Rel_PC), the DT levels of these two types of enterprises are compared, and a t-mean test is conducted. Table 4 reports the results of the t tests based on the grouping of political connections. The DT level of enterprises based on the percentage of sentence words DT and DT_now and whether to carry out digital transformation DT_dummy are significantly different at the 1% level. The mean value of enterprise DT with nonclose political connections (0.074) is significantly greater than that of enterprises with close political connections (0.053), indicating that political connections may negatively impact corporate DT. Next, we use a regression model to conduct a rigorous empirical test.

**Table 4:** Results of the t-means test of variables.

Variables	Observations	Average value	Observations	Average value	Difference of means	t
	Enterprises without political connections		Enterprises with political connections			
*DT*	12277	0.074	6345	0.054	0.021***	15.024
*DT_now*	12277	0.045	6345	0.031	0.014***	14.701
*DT_dummy*	12277	0.898	6345	0.880	0.018***	3.727

Note:

***, **, and * indicate significance levels of 1%, 5% and 10%, respectively.

## 4. Empirical analysis

### 4.1 Model specification

To test the impact of political connections on the level of private enterprises’ DT, the following benchmark regression model is constructed:

$$DT_{it}=\beta_{1}Rel_{it}+\sum \gamma_{i}Control_{it}+\delta_{i}+\mu_{t}+\varepsilon_{it}$$ (1)

Where the explanatory variable *DT*_*it*_ denotes the level of DT of firm *i* in year *t*, the core explanatory variable *Rel*_*it*_ is the political connections of firm *i* in year *t*, *Control*_*it*_ denotes the other control variables presented in Table 1, *δ*_*i*_ and *μ*_*t*_ denote the individual and time fixed effects, respectively, and *ε*_*it*_ represents random disturbance terms. We use heteroskedasticity-robust standard errors in all regression equations.

### 4.2 Baseline regression results

The regression results are presented in Table 5. Columns (1), (2), and (3) demonstrate the regression models with political connections (Rel_PC) and Rel_PCLevel and Rel_ZD as the explanatory variables, respectively. The results demonstrate that, holding other control variables constant, having close political connections results in an average decrease of approximately 0.004 in the level of corporate DT compared to that of private enterprises without close political connections. There is heterogeneity in the effect of different levels of political connections on corporate DTs. The inhibitory effect decreases as the level of association increases, where the level of political connections at the county level and below (Rel_PCLevel = 1) and at the prefecture level (Rel_PCLevel = 2) have a significant inhibitory effect on corporate DT, and the DT level of private enterprises with close political connections decreases on average by approximately 0.006% and 0.005%, respectively, compared to that of private enterprises without close political connections, holding other control variables constant. For individuals at the provincial-ministerial level and above (Rel_PCLevel = 3), the coefficients are negative but not significant. The degree of close political connections is significantly negatively related to the level of DT. Moreover, for every one percentage point increase in the degree of close political connections, the level of enterprise DT decreases by approximately 0.008 on average. The results of the benchmark regression indicate that political connections have a significantly negative impact on corporate DT, and hypothesis H2 is verified.

**Table 5:** Baseline regression results (I).

	(1)	(2)	(3)
Dependent variables	*DT*	*DT*	*DT*
Rel_PC	-0.004***		
	(0.001)		
Rel_PCLevel = 1		-0.006***	
		(0.002)	
Rel_PCLevel = 2		-0.005**	
		(0.002)	
Rel_PCLevel = 3		-0.002	
		(0.002)	
Rel_ZD			-0.008***
			(0.002)
Size	0.010***	0.010***	0.011***
	(0.001)	(0.001)	(0.001)
Lnage	-0.010	-0.010	-0.021*
	(0.009)	(0.009)	(0.011)
Cash	-0.023***	-0.024***	-0.024***
	(0.006)	(0.006)	(0.007)
Lev	-0.021***	-0.022***	-0.026***
	(0.004)	(0.004)	(0.004)
Growth	0.004***	0.004***	0.004***
	(0.001)	(0.001)	(0.002)
ROA	-0.006	-0.006	-0.004
	(0.008)	(0.008)	(0.009)
Top10	-0.016***	-0.016***	-0.011*
	(0.005)	(0.005)	(0.006)
Internet	0.012*	0.012*	0.017**
	(0.007)	(0.007)	(0.008)
Constant	0.032	0.033	0.050
	(0.026)	(0.026)	(0.033)
Firm fixed effects	YES	YES	YES
Time fixed effects	YES	YES	YES
Observations	18622	18622	15484
Adjust R^2^	0.010	0.010	0.013

Note:

***, **, and * indicate significance at the 1%, 5% and 10% levels, respectively. Heteroskedasticity-robust standard errors are included in parentheses. The same is true for Tables 6–14.

**Table 6:** Baseline regression results (II).

	(1)	(2)	(3)	(4)	(5)	(6)
Estimated model	*FE*	*FE*	*FE*	*Logit*	*Logit*	*Logit*
Dependent variables	*DT_now*	*DT_now*	*DT_now*	*DT_dummy*	*DT_dummy*	*DT_dummy*
*Rel_PC*	-0.002**			-0.202*		
	(0.001)			(0.106)		
*Size*	0.007***	0.007***	0.008***	0.466***	0.462***	0.451***
	(0.001)	(0.001)	(0.001)	(0.070)	(0.071)	(0.092)
*Lnage*	0.002	0.002	0.000	-0.158	-0.161	-1.265
	(0.006)	(0.006)	(0.008)	(0.702)	(0.702)	(0.956)
*Cash*	-0.013***	-0.013***	-0.015***	-0.911*	-0.945**	-0.774
	(0.004)	(0.004)	(0.005)	(0.470)	(0.471)	(0.570)
*Lev*	-0.013***	-0.013***	-0.016***	-0.834***	-0.859***	-1.065***
	(0.003)	(0.003)	(0.003)	(0.274)	(0.274)	(0.337)
*Growth*	0.003***	0.003***	0.003***	0.104	0.102	0.192*
	(0.001)	(0.001)	(0.001)	(0.093)	(0.093)	(0.112)
*ROA*	-0.011*	-0.011*	-0.010	0.319	0.310	0.168
	(0.005)	(0.005)	(0.006)	(0.506)	(0.507)	(0.612)
*Top10*	-0.025***	-0.025***	-0.023***	0.072	0.031	0.040
	(0.004)	(0.004)	(0.004)	(0.393)	(0.394)	(0.497)
*Internet*	0.005	0.005	0.008	0.722	0.778	0.291
	(0.005)	(0.005)	(0.005)	(0.591)	(0.594)	(0.699)
*Rel_PCLevel = 1*		-0.003**			-0.325**	
		(0.001)			(0.154)	
*Rel_PCLevel = 2*		-0.002			-0.367**	
		(0.001)			(0.166)	
*Rel_PCLevel = 3*		-0.001			0.036	
		(0.001)			(0.150)	
*Rel_ZD*			-0.004**			-2.784
			(0.002)			(1.973)
*Constant*	-0.001	-0.000	-0.004			
	(0.018)	(0.018)	(0.022)			
Firm fixed effects	YES	YES	YES	YES	YES	YES
Time fixed effects	YES	YES	YES	YES	YES	YES
Observations	18622	18622	15484	7368	7368	5218
Adjust R^2^	0.011	0.011	0.011	-	-	-

**Table 7:** Robustness test results (I).

	(1)	(2)
Dependent variables	*DT_num*	*DT_mda*
*Rel_PC*	-0.002**	-0.033*
	(0.001)	(0.020)
*Size*	0.011***	0.274***
	(0.001)	(0.016)
*Lnage*	0.004	0.131
	(0.007)	(0.133)
*Cash*	-0.015***	-0.385***
	(0.005)	(0.109)
*Lev*	-0.009***	-0.297***
	(0.003)	(0.061)
*Growth*	0.003**	0.068***
	(0.001)	(0.022)
*ROA*	-0.004	0.026
	(0.006)	(0.110)
*Top10*	-0.021***	0.142*
	(0.004)	(0.082)
*Internet*	0.015***	0.138
	(0.005)	(0.109)
*Constant*	-0.026	0.090
	(0.020)	(0.404)
Firm fixed effects	YES	YES
Time fixed effects	YES	YES
Observations	18622	18483
Adjust R^2^	0.021	0.035

**Table 8:** Robustness test results (II).

	(1)	(2)
Dependent variables	*DT*	*DT*
*Rel_PC*	-0.003*	-0.004**
	(0.001)	(0.002)
*Size*	0.010***	0.010***
	(0.001)	(0.001)
*Lnage*	-0.011	-0.011
	(0.009)	(0.009)
*Cash*	-0.023***	-0.023***
	(0.006)	(0.006)
*Lev*	-0.021***	-0.021***
	(0.004)	(0.004)
*Growth*	0.004***	0.004***
	(0.001)	(0.001)
*ROA*	-0.006	-0.006
	(0.008)	(0.008)
*Top10*	-0.016***	-0.016***
	(0.005)	(0.005)
*Internet*	0.012*	0.013*
	(0.007)	(0.007)
*Constant*	0.033	0.034
	(0.026)	(0.026)
Firm fixed effects	YES	YES
Time fixed effects	YES	YES
Observations	18622	18619
Adjust R^2^	0.010	0.010

**Table 9:** Robustness test results (III).

	(1)	(2)	(3)	(4)	(5)
Dependent variables	*DT*	*DT*	*DT*	*DT*	*DT*
*Rel_PC*	-0.004***	-0.002*	-0.004***	-0.004***	-0.004***
	(0.001)	(0.001)	(0.001)	(0.002)	(0.001)
*Size*	0.010***	0.004***	0.010***	0.011***	0.010***
	(0.001)	(0.001)	(0.001)	(0.001)	(0.001)
*Lnage*	-0.009	-0.012	-0.017*	-0.018*	-0.017*
	(0.010)	(0.008)	(0.009)	(0.011)	(0.009)
*Cash*	-0.031***	-0.017***	-0.022***	-0.028***	-0.027***
	(0.008)	(0.006)	(0.007)	(0.008)	(0.007)
*Lev*	-0.023***	-0.008**	-0.022***	-0.025***	-0.024***
	(0.005)	(0.003)	(0.004)	(0.005)	(0.004)
*Growth*	0.004**	0.002*	0.004**	0.003*	0.004***
	(0.002)	(0.001)	(0.002)	(0.002)	(0.001)
*ROA*	-0.003	-0.010	-0.009	-0.009	-0.002
	(0.009)	(0.007)	(0.008)	(0.009)	(0.008)
*Top10*	-0.017***	-0.005	-0.015***	-0.014**	-0.015***
	(0.006)	(0.005)	(0.006)	(0.006)	(0.006)
*Internet*	0.011	0.006	0.019**	0.019**	0.013*
	(0.008)	(0.006)	(0.008)	(0.009)	(0.007)
*Exe_age*					-0.010
					(0.009)
*Exe_women*					0.008
					(0.007)
*Exe_edu*					0.006***
					(0.001)
*Constant*	0.010***	0.004***	0.043	0.046	0.066
	(0.001)	(0.001)	(0.029)	(0.032)	(0.044)
Firm fixed effects	YES	YES	YES	YES	YES
Time fixed effects	YES	YES	YES	YES	YES
Observations	16589	14997	16583	14299	17274
Adjust R^2^	0.009	0.003	0.011	0.012	0.013

**Table 10:** Instrumental variable estimation results.

	(1)	(2)	(3)	(4)	(5)	(6)
	First-stage	Second-stage	First-stage	Second-stage	First-stage	Second-stage
Dependent variables	*Rel_PC*	*DT*	*Rel_PC*	*DT*	*Rel_PC*	*DT*
*Rel_PC*		-0.144**		-0.007***		-0.007***
		(0.057)		(0.002)		(0.002)
*Rel_PC_AVE(IV1)*	0.217***				0.033***	
	(0.064)				(0.009)	
*L*.*Rel_PC(IV2)*			0.634***		0.630***	
			(0.011)		(0.012)	
*Size*	0.052***	0.017***	0.019***	0.010***	0.015***	0.011***
	(0.007)	(0.003)	(0.006)	(0.001)	(0.006)	(0.001)
*Lnage*	0.157***	0.011	0.084*	-0.015	0.090**	-0.017
	(0.056)	(0.015)	(0.045)	(0.010)	(0.046)	(0.011)
*Cash*	-0.078*	-0.034***	-0.044	-0.021***	-0.033	-0.018**
	(0.043)	(0.010)	(0.036)	(0.007)	(0.037)	(0.007)
*Lev*	-0.033	-0.026***	-0.004	-0.020***	-0.011	-0.020***
	(0.026)	(0.005)	(0.023)	(0.004)	(0.023)	(0.004)
*Growth*	0.002	0.004**	0.008	0.004***	0.008	0.004***
	(0.009)	(0.002)	(0.008)	(0.001)	(0.008)	(0.002)
*ROA*	-0.059	-0.014	-0.030	-0.010	-0.011	-0.009
	(0.047)	(0.011)	(0.040)	(0.008)	(0.040)	(0.009)
*Top10*	-0.024	-0.021***	-0.028	-0.019***	-0.025	-0.021***
	(0.035)	(0.007)	(0.030)	(0.005)	(0.031)	(0.006)
*Internet*	-0.229***	-0.021	-0.103**	0.007	-0.084*	0.005
	(0.048)	(0.017)	(0.043)	(0.007)	(0.045)	(0.008)
Firm fixed effects	YES	YES	YES	YES	YES	YES
Time fixed effects	YES	YES	YES	YES	YES	YES
Sample size	18600	18600	15780	15780	15263	15263
KPF statistic	17.683		3065.166		1457.079	
Stock—Yogo 10% threshold	16.38		16.38		19.93	
Hansen J test p value	-		-		0.19	

**Table 11:** Regression results of alleviating omitted variables.

	(1)	(2)	(3)
	OLS	OLS	2SLS
Dependent variables	*DT*	*DT*	*DT*
*Rel_PC*	-0.004***	-0.004***	-0.007***
	(0.001)	(0.001)	(0.002)
*Size*	0.010***	0.009***	0.010***
	(0.001)	(0.001)	(0.001)
*Lnage*	-0.006	-0.013	-0.022*
	(0.009)	(0.009)	(0.012)
*Cash*	-0.024***	-0.021***	-0.017**
	(0.007)	(0.006)	(0.008)
*Lev*	-0.023***	-0.023***	-0.020***
	(0.004)	(0.004)	(0.004)
*Growth*	0.004***	0.004***	0.005***
	(0.001)	(0.001)	(0.002)
*ROA*	-0.006	-0.003	-0.006
	(0.008)	(0.008)	(0.009)
*Top10*	-0.012**	-0.000	-0.006
	(0.005)	(0.005)	(0.006)
*Internet*	-	-	-
	-	-	-
*Constant*	0.022	0.040	
	(0.027)	(0.027)	
Firm fixed effects	YES	YES	YES
Time fixed effects	YES	YES	YES
Province fixed effects	YES	YES	YES
Province*Time fixed effects	YES	YES	YES
Industry fixed effects	NO	YES	YES
Industry*Time fixed effects	NO	YES	YES
Observations	18622	18619	15263

**Table 12:** Estimation results based on propensity score matching (PSM).

	(1)	(2)	(3)
Matching method	K-nearest neighbor matching	Caliper matching	Kernal matching
Dependent variables	*DT*	*DT*	*DT*
*Rel_PC*	-0.003***	-0.004***	-0.004***
	(0.001)	(0.001)	(0.001)
*Size*	0.009***	0.010***	0.010***
	(0.001)	(0.001)	(0.001)
*Lnage*	-0.007	-0.010	-0.010
	(0.009)	(0.009)	(0.009)
*Cash*	-0.023***	-0.023***	-0.023***
	(0.007)	(0.006)	(0.006)
*Lev*	-0.019***	-0.021***	-0.021***
	(0.004)	(0.004)	(0.004)
*Growth*	0.004***	0.004***	0.004***
	(0.001)	(0.001)	(0.001)
*ROA*	-0.006	-0.005	-0.005
	(0.008)	(0.008)	(0.008)
*Top10*	-0.011**	-0.017***	-0.017***
	(0.006)	(0.005)	(0.005)
*Internet*	0.016**	0.012*	0.012*
	(0.007)	(0.007)	(0.007)
*Constant*	0.021	0.032	0.032
	(0.028)	(0.026)	(0.026)
Firm fixed effects	YES	YES	YES
Time fixed effects	YES	YES	YES
Observations	15605	18464	18464
Adjust R^2^	0.009	0.010	0.010

**Table 13:** Analysis of the influence mechanism of political connections on corporate digital transformation.

	(1)	(2)	(3)	(4)
Dependent variables	*DT*	*Risk_taking*	*RD*	*Burden*
*Rel_PC*	-0.004***	-0.331*	-0.147**	0.051**
	(0.001)	(0.202)	(0.072)	(0.021)
*Size*	0.010***	-1.182***	0.177**	0.028
	(0.001)	(0.297)	(0.069)	(0.020)
*Lnage*	-0.010	7.761***	-1.654***	-0.136
	(0.009)	(2.125)	(0.490)	(0.148)
*Cash*	-0.023***	-1.418	-0.777**	0.013
	(0.006)	(1.637)	(0.359)	(0.121)
*Lev*	-0.021***	5.052***	-2.661***	-0.052
	(0.004)	(1.127)	(0.263)	(0.070)
*Growth*	0.004***	-0.078	-0.700***	-0.024
	(0.001)	(0.335)	(0.097)	(0.027)
*ROA*	-0.006	-16.401***	-7.596***	-0.121
	(0.008)	(2.572)	(0.536)	(0.123)
*Top10*	-0.016***	-2.350*	0.766**	0.264**
	(0.005)	(1.263)	(0.320)	(0.104)
*Internet*	0.012*	0.280	0.936**	0.136
	(0.007)	(1.575)	(0.409)	(0.115)
*Constant*	0.032	-6.960	8.919***	0.563
	(0.026)	(6.387)	(1.518)	(0.462)
Firm fixed effects	YES	YES	YES	YES
Time fixed effects	YES	YES	YES	YES
Observations	18622	16144	16053	15760
Adjust R^2^	0.010	0.233	0.056	0.003

**Table 14:** Heterogeneity analysis.

	(1)	(2)	(3)	(4)	(5)	(6)	(7)	(8)
	Weakly competitive	Strong Competitive	Low Growth	High Growth	Non-high-tech	High Tech	Change in municipal officers	No change in municipal officers
Dependent variables	*DT*	*DT*	*DT*	*DT*	*DT*	*DT*	*DT*	*DT*
*Rel_PC*	-0.002	-0.004**	-0.003**	-0.003	-0.004**	-0.002	-0.008**	-0.004**
	(0.002)	(0.002)	(0.002)	(0.002)	(0.002)	(0.002)	(0.003)	(0.002)
*Size*	0.004***	0.014***	0.011***	0.007***	0.008***	0.011***	0.012***	0.013***
	(0.001)	(0.001)	(0.001)	(0.002)	(0.002)	(0.001)	(0.003)	(0.002)
*Lnage*	0.005	-0.032**	-0.011	-0.016	0.033**	-0.035***	-0.025	-0.007
	(0.011)	(0.013)	(0.011)	(0.019)	(0.013)	(0.012)	(0.024)	(0.017)
*Cash*	-0.007	-0.033***	-0.016**	-0.026*	-0.013*	-0.032***	-0.003	-0.011
	(0.008)	(0.010)	(0.008)	(0.014)	(0.008)	(0.009)	(0.018)	(0.011)
*Lev*	-0.001	-0.037***	-0.020***	-0.025***	-0.013**	-0.019***	-0.028**	-0.036***
	(0.005)	(0.006)	(0.005)	(0.008)	(0.005)	(0.005)	(0.011)	(0.007)
*Growth*	0.004**	0.003	0.001	0.005*	0.004**	0.004**	0.010***	0.004
	(0.002)	(0.002)	(0.003)	(0.003)	(0.002)	(0.002)	(0.004)	(0.002)
*ROA*	-0.009	-0.002	-0.016*	0.022	-0.005	-0.006	-0.014	-0.020
	(0.009)	(0.012)	(0.009)	(0.017)	(0.011)	(0.010)	(0.020)	(0.015)
*Top10*	0.013**	-0.031***	-0.019***	-0.020*	0.003	-0.028***	-0.024	-0.009
	(0.006)	(0.008)	(0.006)	(0.012)	(0.007)	(0.008)	(0.015)	(0.009)
*Internet*	0.012	0.020**	0.008	0.021	0.026***	0.002	0.001	0.026**
	(0.009)	(0.010)	(0.008)	(0.015)	(0.010)	(0.009)	(0.020)	(0.012)
*Constant*	-0.014	0.086**	0.029	0.069	-0.101**	0.101***	0.064	-0.010
	(0.033)	(0.040)	(0.034)	(0.057)	(0.040)	(0.035)	(0.071)	(0.048)
Firm fixed effects	YES	YES	YES	YES	YES	YES	YES	YES
Time fixed effects	YES	YES	YES	YES	YES	YES	YES	YES
Observations	7938	10509	12209	5834	7563	10865	2873	6222
Adjust R^2^	0.005	0.018	0.011	0.009	0.011	0.011	0.023	0.018

We further use the level of DT defined as how well a firm is currently using digital technologies (DT_now) and whether it is undertaking digital transformation (DT_dummy) as explanatory variables; the regression results are reported in Table 6. Columns (1) to (3) reflect the use of DT_now as the explanatory variable, which is regressed using a panel fixed effects model. Columns (4) to (6) reflect the use of the dummy variable DT_dummy as the explanatory variable, which is regressed using the panel logit model. The results demonstrate that the core explanatory variable Rel_PC is significantly negative in both regression models. Among the political connection-level variables, the effect at the county level and below (Rel_PCLevel = 1) remains the most significant, and the coefficient at the prefecture level (Rel_PCLevel = 2) is significantly negative in the panel logit model and negative but not significant in the panel fixed effects model. The degree of close political connection (Rel_ZD) negatively affects DT_now but does not significantly affect whether firms undertake DT (DT_dummy).

### 4.3 Robustness test

To ensure that the conclusions are robust and reliable, a series of robustness tests are conducted. First, the metric of the DT level of the explanatory variables is changed. Two methods are used to reconstruct the measure of the DT level. First, sentence frequency data are used to replace sentence word count share data [44]. The number of sentences that imply that private enterprises are using or will use the six types of digital technologies as a proportion of the total number of sentences in their annual reports (DT_num) is used as an alternative variable for the level of enterprise DT. Second, word frequency data are used instead of sentence word count shares [44]. Keywords related to DT are identified, and the frequency with which words appear in the MD&A section of the annual reports of listed companies is counted. The natural logarithm (DT_mda) is used as an alternative variable for DT. Table 7 reports the test results. The coefficients of the explanatory variables measuring political connections are all significantly negative-at least at the 10% significance level-compared to the results of the benchmark regression, indicating that these results are robust.

Then, we change the measure of the explanatory variable corporate political connections (Rel). We use two methods to remeasure corporate political connections. First, as the general manager is primarily responsible for the firm’s decisions regarding daily management and affairs, his or her political status is used as an alternative measure of corporate political connections [18, 19]. Second, in addition to chairpersons and general managers, independent directors serving in government departments are also important political resources for enterprises [48], as they can effectively assist in corporate decision making and significantly improve business performance and governance [46]. Therefore, we use the proportion of independent directors with close political connections to all independent directors as an alternative measure of corporate political connections [14]. Table 8 reports the results of the test. The level of DT is lower in firms whose general managers have close political connections and with a greater proportion of independent directors with close political connections, which implies that the findings of the study remain unchanged.

Third, special samples are excluded. The DT indicators constructed in this study may be affected by the heterogeneous behaviors of some enterprises. Some enterprises may disclose selective information in their annual reports, causing to biased judgments in their DT behaviors. Subsequently, enterprises with a DT of 0 for the DT level are excluded. Moreover, to eliminate the influence of some enterprises characterized by “natural digitalness,” a sample of enterprises closely related to digital technology adoption is excluded. Industries related to the adoption of digital technology include information transmission, software and information technology services, computer, communications and other electronic equipment manufacturing in the manufacturing industry, and scientific research and technical services in the science and technology promotion and application services. The results in Columns (1) and (2) of Table 9 illustrate that the estimated coefficient of the political connections variable Rel_PC is still significantly negative, at least at the 10% level, after excluding the special sample. In addition, considering two major exogenous event shocks during the sample period of this study—the 2008 global financial crisis and the 2015 Chinese stock market crash—when facing major exogenous market shocks, firms’ production and operations are severely affected, and DT decisions and implementation may be hindered. To eliminate this influence, we exclude samples from 2008 and 2015. In addition, considering the time lag of the impact of exogenous shocks, we further exclude samples from 2008, 2009, 2015 and 2016 [49]. The results in Columns (3) and (4) of Table 9 illustrate that the estimated coefficient of the political connections variable Rel_PC is still significantly negative at the 1% level.

Fourth, given that the measurement of political connections is based on whether company executives, chairpersons or general managers have served or are serving on party committees and in governments, individuals might be willing to serve in such institutions if they inherently exhibit a more conservative outlook and adopt a cautious stance toward new technologies. To rule out this influence, we incorporate in the baseline regression control variables reflecting executive attributes, including executives’ average age (*Exe_age*), proportion of female executives (*Exe_women*) and average level of education (*Exe_edu*). The results in Column (5) of Table 9 illustrate that the estimated coefficient of the political connections variable Rel_PC is still significantly negative at the 1% level. The above results indicate that the conclusions of this study are robust.

### 4.4 Managing endogeneity

#### 4.4.1 Reverse causality problem

The findings of the baseline regression model in this study may be affected by the reverse causality problem, where corporate DTs may, in turn, affect the construction of corporate political connections, leading to bias in the model estimation results. To mitigate the impact of this problem on the findings as much as possible, we construct an instrumental variable of political connections and select Rel_PC_AVE, which is the proportion of enterprises with close political connections other than the enterprise in the same region and the same industry, as the instrumental variable of Rel_PC [3]. This instrumental variable is rational in two respects. First, the external environment in which a firm is located has an important influence on its behavior. When enterprises in the same industry or region establish political connections, they usually tend to build political connections for market competition and risk avoidance; thus, individual enterprises’ level of political connections is related to the overall level of political connections in the same region and industry, which meets the relevance requirement of the instrumental variable. Second, the characteristic variables of the overall sample do not directly affect individual firm behavior, and the political connection situation of firms other than individual firms is not directly related to their DT level; thus, the instrumental variables satisfy the exogeneity requirement.

In addition, we use the first-order lagged term L.Rel_PC of the core explanatory variable, Rel_PC, as an additional instrumental variable [50]. Table 10 reports the results of the two-stage least squares regression of the instrumental variables. The results demonstrate that the political connection variable Rel_PC is significantly positively correlated with both instrumental variables and has a significant inhibitory effect on the level of private firms’ DTs. Moreover, the Kleibergen‒Paap rk Wald F statistic is greater than the Stock—Yogo 10% critical value, indicating that the model does not have a weak instrumental variable problem; the Hansen J statistic also passes the identification test, indicating that the instrumental variable does not have an overidentification problem. The estimation results of the instrumental variables indicate that close government—business relationships are detrimental to corporate DT, and the findings of the benchmark regression are further validated.

#### 4.4.2 Omitted variables issues

The baseline model may miss some variables that affect both corporate DT and political connections. To alleviate this issue, we further control for high-order fixed effects, including province×time fixed effects and industry×time fixed effects, in the baseline regression and two-stage least squares regression. Table 11 shows that the coefficient of the explanatory variable is still significant.

#### 4.4.3 Sample selection problem

To rule out possible endogeneity problems due to sample self-selection, the PSM method was used for testing. We group the full sample according to whether firms have close political connections and match them with the control variables in the benchmark regression, industry and year as covariates. Table 12 reports the estimation results after matching, where the explanatory variables Rel_PC are all significantly negative, at least at the 1% level, and the regression coefficients are closer to the baseline regression results.

## 5. Further discussion

### 5.1 Examination of the mechanism through which political connections inhibit corporate digital transformation

Both the fixed effect-based baseline regression estimation and the robustness and endogeneity test results indicate that close political connections have an inhibitory effect on corporate DTs. By examining the relevant literature at home and abroad, we analyze and test the mechanisms by which political connections may affect corporate DTs. Starting from the three major elements of enterprise DT—entrepreneurs, technology, and resources—we propose three major effects of political connections that inhibit corporate DTs. To ensure the completeness of empirical analysis, we also examine the potential positive mechanism in S1 File.

First, there is a risk-weakening effect. From the entrepreneur’s perspective, close political connections make business managers short-sighted and weaker in their willingness and ability to take risks, resulting in less incentive to undertake DTs. Risk-taking is an important decision for private entrepreneurs in production operations. A higher level of risk-taking implies that firms are willing to invest resources in plans with unknown outcomes [51]. DT implies a change in a company’s traditional production and operational model, and the resulting increased costs and risks must be considered when making decisions. The benefits of DT may be more of a spillover effect in the short term, with little benefit to the company itself and a high risk of failure, thus creating a “digital paradox”. To test hypothesis H2a, we use ROA volatility to measure corporate risk-taking [52]. Specifically, Eqs (2) and (3), which measure firms’ risk-taking levels, are used to calculate the industry-adjusted volatility of firms’ ROA over 3 years.


$${Risk\_taking}_{in}=\sqrt{\frac{1}{N-1}\sum_{n=1}^{N}{\left({ADJ}_{{ROA}_{in}}-\frac{1}{N}\sum_{n=1}^{N}{ADJ}_{{ROA}_{in}}\right)}^{2}}$$ (2)

$$ADJ_{ROA_{in}}=ROA_{in}-\frac{1}{X_{n}}\sum_{k=1}^{X}ROA_{kn}$$ (3)

Table 13 reports the results. Column (1) of Table 13 presents the estimation results of the baseline regression model (1). Column (2) reports the test results for political connections and corporate risk-taking, demonstrating that close political connections (Rel_PC) have a significant negative effect on corporate risk-taking and weaken the risk-taking willingness of private enterprises and negatively impact their DTs.

Second, there is an innovation inhibition effect. From a technological perspective, political connections have an inhibitory effect on the technological innovation of private enterprises. Enterprises’ successful DTs cannot be achieved without technological innovation. However, as private firms’ political and business relationships become closer, they are more likely to rely on political resources rather than maintaining competitiveness by improving their technological innovation capabilities. In addition, political connections can lead to financial misallocation [53], thus increasing investments in nonproductive activities and reducing funding sources for corporate innovation, which constrains firms’ innovation activities.

To test hypothesis H2b, we investigate the impact of political connections on innovation activities by measuring the level of corporate innovation investment (RD) as the share of total corporate R&D expenditure in operating revenue. Column (3) of Table 13 reports the results of the test between political connections and firm innovation; the regression coefficient of close political connections (Rel_PC) on innovation investment level RD is negative and significant at the 5% level, indicating that close political connections significantly reduce private firms’ innovation investment levels, thus inhibiting corporate DTs.

Third, there is a resource crowding-out effect. From the perspective of resources, political connections consume substantial resources from private enterprises [29, 30], which has a “crowding-out” effect on corporate DTs. Generally, corporate DTs require significant investments [54], including technological innovation, talent recruitment and training, and the acquisition of digital facilities [52]. Although some local governments have provided some support for DTs, the high cost of such transformations means that many enterprises still face the dilemma of “not being able to switch”. Close to 70% of private enterprises state that their own financial constraints, lack of talent, and limited investment capacity limit further development of DT.

To test hypothesis H2c, we choose policy burdens to examine the resource-crowding-out of private firms through political connections. The government may require private enterprises with political connections to increase employment, taxation, and social stability. For example, there are usually excess employees in politically connected enterprises, thus imposing a policy burden on them, which increases the depletion of private enterprises’ resources. Eq 4 is used to calculate the intensity of the policy burden (Burden) for firms based on their optimal capital intensity:

$$Capital_{it}=\alpha_{0}+\alpha_{1}Size_{i\;t-1}+\alpha_{2}Lev_{i\;t-1}+\alpha_{3}ROA_{i\;t-1}+\alpha_{4}Growth_{i\;t-1}+\alpha_{5}Tangi_{i\;t-1}+\varepsilon_{it}$$ (4)

where *Capital*_*it*_ denotes capital intensity, obtained by dividing net fixed assets by the number of employees, and the other control variables are firm size, leverage, return on total assets, revenue growth rate, and asset structure, controlling for region, industry, and time fixed effects. A positive residual of Eq 2 (*ε*) indicates the enterprise’s strategic burden; when it is negative, this indicates the social burden. Moreover, the larger the absolute value of the residual is, the heavier is the policy burden borne by the enterprise. Column (4) of Table 13 reports the test results for political connections and enterprise resource crowding-out. The regression coefficient of close political connections (Rel_PC) in Column (4) is significantly positive, indicating that political connections increase the policy burden on private enterprises and crowd out enterprise resources, which also affects corporate DTs.

According to the above empirical test results, political connections weaken private entrepreneurs’ willingness to take risks, enterprise innovation input‒output decreases, and the degree of resource appropriation increases, which indicates that risk weakening, innovation inhibition, and resource crowding-out effects are indeed the three mechanisms through which political connections inhibit private enterprises’ DTs.

### 5.2 Heterogeneity analysis

We examine the heterogeneous inhibitory effect of political connections on corporate DTs from the perspectives of firm competitiveness, revenue growth, differences in industry attributes, and the replacement of municipal party secretaries in prefecture-level cities.

#### 5.2.1 Industry competitiveness

From the perspective of competition, in less competitive industries, enterprises face less competitive pressure and have more “voice” in the industry; however, these enterprises usually have richer human and financial resource reserves, and their “bargaining power” in the face of government requirements is also greater. Therefore, the degree of inhibition of corporate DT may be weaker in less than more competitive industries. Based on whether the Herfindahl index of private enterprises’ industries exceeds the median for their industries in the current year, the sample enterprises are divided into two categories—strongly and weakly competitive enterprises—and regressed according to the benchmark regression model (1). Table 14 reports the results. Columns (1) and (2) of Table 14 demonstrate that the coefficient of the political connections variable (Rel_PC) is -0.004 and significant at the 5% level in the group of strongly competitive firms, and the coefficient of Rel_PC is negative but not significant in the group of weakly competitive firms, which is consistent with the analysis in this study.

#### 5.2.2 Business growth

From the perspective of enterprise business revenue growth, the lower the revenue growth rate is, the greater the internal management costs are, and the greater the inhibitory effect of digital transformation is expected to be under the influence of the lagged management model of executives in enterprises with political connections. The sample firms are divided into low- and high-growth firms based on whether the growth rate of private firms’ business revenue exceeds the median in their industries in the current year and is regressed according to benchmark regression Eq 1. The results in Columns (3) and (4) of Table 14 demonstrate that the coefficient of Rel_PC is negative and significant at the 5% level for the group of low-growth firms, whereas for the group of high-growth firms, the coefficient of Rel_PC is negative but not significant, indicating that the inhibitory effect of political connections on the DT of low-growth private firms is more pronounced.

#### 5.2.3 Industry differences

The level of DT development varies significantly among industries, and the market environments faced by different industries also differ. Thus, interindustry differences may exist in the impact of political connections on corporate DT. High-tech firms depend more on innovation activities; thus, DT is a key area of attention and investment. Moreover, high-technology firms are supported more by central and local government industrial policies and are more likely to receive government subsidies. Therefore, the inhibitory effect of political connections on private enterprises’ DT may be more pronounced among non-high-tech enterprises. According to the National Bureau of Statistics Classification of High Technology Industries (Manufacturing) (2017) and the National Statistical Classification of Science and Technology Services (2018), the sample enterprises were divided into high-tech and non-high-tech enterprises, and their heterogeneous effects were compared. The results in Columns (5) and (6) of Table 14 demonstrate that the coefficient of Rel_PC is significantly negative for the group of non-high-tech enterprises, and the regression coefficient of Rel_PC for the high-tech enterprise group is negative and nonsignificant, indicating that the inhibitory effect of political connections on the DT of non-high-tech private enterprises is greater than that for high-tech enterprises.

#### 5.2.4 City secretary replacement

The benchmark regression demonstrates the significant negative impact of county- and prefecture-level political and business relations on corporate DT. Municipal party secretaries, as the “handlers” of prefecture-level municipalities, have a greater influence on local economic development and policy implementation. If municipal party secretaries in the prefecture-level municipalities where private enterprises are located change, enterprises spend more resources seeking new political and business relationships, and the crowding-out effect on DT may be stronger. As it takes some time from when municipal party secretaries take office to when they have a real impact on the economic development of the prefecture-level city in which they are located, if a new municipal party secretary takes office from January to June of the current year, the current year is defined as the change year; otherwise, the next year is defined as the change year. The results in Columns (7) and (8) of Table 14 demonstrate that the absolute values of Rel_PC regression coefficients are higher for the samples in which municipal party secretaries changed in the year during which firms are at the prefecture level than for the samples without a change in the number of municipal party secretaries, indicating that a change in the number of municipal party secretaries increases the inhibitory effect of political connections on corporate DT.

## 6. Conclusions

Given the accelerated development of the digital economy, DT has changed from an “optional” to a “mandatory” option for enterprises. We analyze the specific impact of political connections on corporate DT and its mechanisms of action using data from privately listed A-share firms in Shanghai and Shenzhen from 2008 to 2020 as the research sample. The annual reports disclosed by the listed enterprises were used as text data, and DT metrics were constructed using a supervised machine learning method. The political connections of enterprises are measured by the political career experience of chairpersons and managers and the proportion of in-service consumption expenditure to business revenue, and the mechanism of the influence of political connections on corporate DT is explored. The empirical test results demonstrate that close political connections have a significant inhibitory effect on corporate DTs, and these findings hold after a series of robustness and endogeneity tests. The results of the mechanism analysis indicate that political connections inhibit corporate DTs primarily through three mechanisms: weakening risk, inhibiting innovation, and resource crowding-out effects.

A heterogeneity analysis was also conducted from the perspectives of firm competitiveness, revenue growth, industry attributes, and whether the municipal party secretary of the prefecture in which the firm is located was replaced. The results demonstrate that the inhibitory effect of political connections on corporate DTs is more pronounced among strongly competitive firms, firms with lower revenue growth, non-high-tech firms, and firms in which the municipal party secretary of the prefecture in which the firm is located is replaced in the current year.

This article has positive theoretical and practical significance. From theoretical perspective, we expand the literature on the impact of political connections on firms and further enrich the research on the factors influencing enterprise digital transformation. From practical perspective, the conclusions of this article imply that to accelerate corporate DTs, decentralization should be promoted. Cordial and clean government—business relationships should be built. The central position of enterprises in DTs should be strengthened. Interventions in the planning, production, and management of private enterprises should be reduced. And the vitality of corporate DTs should be stimulated.

## Supporting information

S1 File Empirical analysis of the potential positive influence of political connections on enterprise DT. (DOCX)

S1 Data (XLSX)
