# Supplementary material for: Do political connections facilitate or inhibit firms’ digital transformation? Evidence from China’s A-share private listed companies
Source: PLoS One. 2024 May 7;19(5):e0302586. doi: 10.1371/journal.pone.0302586 (PMC11075905; doi:10.1371/journal.pone.0302586)
Supplement: S1 File — (DOCX) [file pone.0302586.s001.docx]

**Supporting information**

S1 File. Empirical analysis of the potential positive influence of political connections on enterprise DT

While the overall impact appears negative, it does not necessarily imply the absence of positive influences. To verify possible positive mechanisms, we test hypotheses H1a and H1b in the body of the article.

First, we use the proportion of subsidies received by enterprises to total income (Sub) and the SA financing constraint index (SA_index) to measure the financial support of enterprises. The results of Columns (1) and (2) in Table S1 show that political connections can alleviate financing constraints for enterprises but cannot significantly increase the subsidies they receive.

Second, we use enterprises’ actual earnings management situation (DA) to measure their information asymmetry. The results of Column (3) in Table S1 show that political connections do not significantly improve information asymmetry in enterprises.

**Table S1. Analysis of the potential positive effect of political connections on corporate digital transformation.**

|  | (1) | (2) | (3) |
| --- | --- | --- | --- |
| Dependent variables | *Sub* | *SA_index* | *DA* |
| *Rel_PC* | -0.001 | -0.011*** | -0.001 |
|  | (0.000) | (0.003) | (0.002) |
| *Size* | -0.000 | -0.056*** | -0.004*** |
|  | (0.000) | (0.004) | (0.001) |
| *Lnage* | 0.001 | -0.134*** | 0.026** |
|  | (0.003) | (0.021) | (0.011) |
| *Cash* | -0.005** | 0.022 | -0.071*** |
|  | (0.002) | (0.029) | (0.013) |
| *Lev* | -0.000 | 0.111*** | 0.046*** |
|  | (0.001) | (0.024) | (0.006) |
| *Growth* | -0.006*** | -0.023*** | 0.014*** |
|  | (0.001) | (0.005) | (0.002) |
| *ROA* | 0.001 | 0.019 | -0.128*** |
|  | (0.003) | (0.041) | (0.016) |
| *Top10* | 0.004** | 0.089*** | 0.027*** |
|  | (0.002) | (0.016) | (0.007) |
| *Internet* | 0.006*** | 0.002 | -0.015* |
|  | (0.002) | (0.013) | (0.009) |
| *Constant* | 0.008 | -2.641*** | 0.003 |
|  | (0.008) | (0.060) | (0.034) |
| Firm fixed effects | YES | YES | YES |
| Time fixed effects | YES | YES | YES |
| Observations | 18031 | 18622 | 15871 |
| Adjust R^2^ | 0.233 | 0.056 | 0.003 |
